# Psychoactive Substance Use among Second-Year and Third-Year Medical Students of a Medical College: A Descriptive Cross-sectional Study

**DOI:** 10.31729/jnma.6525

**Published:** 2021-06-30

**Authors:** Alisha Sapkota, Vinutha Silvanus, Priyanka Shah, Sanjeev Chandra Gautam, Anjeel Chhetri

**Affiliations:** 1 Nepal Medical College and Teaching Hospital, Kathmandu, Nepal; 2 Department of Community Medicine, Nepal Medical College and Teaching Hospital, Kathmandu, Nepal; 3 Department of Psychiatry, Nepal Medical College and Teaching Hospital, Kathmandu, Nepal; 4 Department of Psychiatry, Devdaha Medical College, Devdaha, Nepal

**Keywords:** *alcohol*, *cannabis*, *medical students*, *smoking*, *substance use*

## Abstract

**Introduction:**

Psychoactive substance use among medical students is common. This may not only pose a threat to their health and academic performance but may have medico-legal and ethical ramifications. The aim of this study was to find out the prevalence of six psychoactive substances (alcohol, tobacco, cannabis, cocaine, benzodiazepines, opioids) among second year and third year medical students.

**Methods:**

A descriptive cross-sectional study was done in a medical college. Whole sampling was done and ethical approval was taken from the Institutional Review Committee (Reference Number: 54-074/075). The study was conducted from May 2018 to June 2018. A semi-structured self-administered questionnaire modified and adapted from World Health Organization's guidelines for student substance use survey was used to collect data from second year and third year medical students. Statistical Package for Social Sciences version 16.0 was used for analysis. Point estimate at 95% Confidence Interval was calculated along with frequency and proportion for binary data.

**Results:**

Out of 226 total respondents, 95 (42.0%) (35.55-48.45 at 95% Confidence Interval) reported current use of one or more psychoactive substances. Most frequently used substance was alcohol with current use prevalence of 87 (38.5%), followed by smoking 39 (17.3%) and cannabis 27 (11.9%). Cocaine, benzodiazepines and opioids were the least consumed substances with current use prevalence of 2 (0.9%) each.

**Conclusions:**

Almost half of the students were currently using one or more psychoactive substances which is concerning, and therefore strategies must be adopted to alleviate such use.

## INTRODUCTION

The World Health Organization (WHO) defines psychoactive substances as, "substances that, when taken in or administered into one's system, affect mental processes, e.g. perception, consciousness, cognition or mood and emotions."^1^ Psychoactive substance use has been increasing globally, owing to the modification in lifestyles and increasing acceptance of use of such substances in general public.^2^

Psychoactive substance use is common among medical students.^2^ Recurrent and prolonged use may ultimately result in abuse and dependency.^3^ This can pose a negative impact on the health and academic performance of students, while also having medico-legal and ethical consequences.^4,5^

The aim of this study is to find out the prevalence of psychoactive substance use among second year and third year medical students. This study focuses on six types of psychoactive substances like- alcohol, tobacco, cannabis, cocaine, benzodiazepines and opioids.

## METHODS

This was a descriptive cross-sectional study done on second and third year students of a medical college located in Kathmandu Valley from May 2018 to June 2018, using the whole sampling method. Ethical approval was taken from the Research and Institutional Review Committee (IRC) of NMCTH (Reference Number: 54-074/075).

The minimum sample size required for the study was calculated as follow:

$$\begin{array}{ll} \text{n} & \text{= }{\text{Z}}^{\text{2}}\times (\text{p}\times \text{q)}/{\text{e}}^{\text{2}}\\ & \text{= }{\left(1.96\right)}^{\text{2}}\times 0.5\times 0.5/{(\text{0.07)}}^{\text{2}}\\ & \text{= }196 \end{array}$$

where,

n = minimum sample size
Z = 1.96 at 95% Confidence Interval (CI)
p = prevalence taken as 50% for maximum sample size
q = (1-p)
e = margin of error, 7%

Total sample size was calculated to be 196. Adding 10% non respondence rate the sample size becomes 216. However we took a sample size of 226 in our study.

A semi-structured self-administered questionnaire modified and adapted from WHO's guidelines for student substance use survey was used.^6^ Before being finalized, the questionnaire was also pretested on a group of medical students who were not part of the actual survey. The questionnaire included information on prevalence of lifetime substance use (any use during a person's life), use in the last 12 months (recent use) and use in the last 30 days (current use) of six psychoactive substances: alcohol, tobacco, cannabis, cocaine, benzodiazepines and opioids. It included information on socio demographic factors including age, sex, year of study, current place of residence and academic performance in medical school. Academic performance was assessed based on students' own rating. Second and third year medical students were contacted after their class hours, and were invited to participate in the survey. Verbal informed consent was taken from all the students participating in the survey. Assurance regarding confidentiality of the responses was provided and maintained. The students were allowed to ask questions in order to clarify any confusion or misunderstanding. After completing the questionnaire, the participants were asked to put the filled questionnaire in a box placed in front of the classroom. Out of 250 questionnaires distributed, 226 were received with response rate of 90.4%. The terms 'psychoactive substance use' and 'substance use' have been interchangeably used in this research.

Data entry and analysis was done using Statistical Package for the Social Sciences version 16. Statistical tools such as frequency, percentage, median and interquartile range and Confidence Interval at 95% were used.

## RESULTS

Among the 226 respondents, 95 (42.0%) (35.55-48.45 at 95% CI) respondents reported current use (use in the last 30 days) of one or more substances. Seventy-one (60.2%) males and 24 (22.2%) females reported current use. The most currently used substance was alcohol with current use prevalence of 87 (38.5%) followed by tobacco 39 (17.2%) and cannabis 27 (11.9%). Cocaine, benzodiazepine and opioid were the least consumed substances, with current use prevalence being 2 (0.9%). Three (1.3%) students reported recent use of cocaine (use in the last one year); two (0.9%) were found to be using it currently and both were females (Table 1).

**Table 1:** Prevalence of substance use among the respondents.

	Lifetime Alcohol use n (%)	Lifetime Tobacco use n (%)	Lifetime Cannabis use n (%)	Lifetime Cocaine use n (%)	Lifetime Benzodiazepine use n (%)	Lifetime Opioid use n (%)
**Males (n= 118)**	95 (80.5)	54 (45.8)	35 (29.7)	1 (0.8)	2 (1.7)	1 (0.8)
**Females (n=108)**	67 (62.0)	9 (8.3)	7 (6.5)	2 (1.9)	1 (0.9)	2 (1.9)
**Total (n=226)**	162 (71.7)	63 (27.9)	42 (18.6)	3 (1.3)	3 (1.3)	3 (1.3)
	**Recent Alcohol use n (%)**	**Recent Tobacco use n (%)**	**Recent Cannabis use n (%)**	**Recent Cocaine use n (%)**	**Recent Benzodiazepine use n (%)**	**Recent Opioid use n (%)**
**Males (n=118)**	88 (74.6)	49 (41.5)	32 (27.1)	1 (0.8)	2 (1.7)	1 (0.8)
**Females (n=108)**	60 (55.6)	8 (7.4%)	6 (5.6)	2 (1.9)	1 (0.9)	1 (0.9)
**Total (n=226)**	14S (65.5)	57 (25.2)	38 (16.8)	3 (1.3)	3 (1.3)	2 (0.9)
	**Current Alcohol use**	**Current Tobacco use**	**Current Cannabis**	**Current Cocaine**	**Current Benzodiazepine**	**Current Opioid use**
	**n (%)**	**n (%)**	**use n (/)**	**use n (/)**	**use n (/)**	**n (%)**
**Males (n= 118)**	63 (53.4)	38 (32.2)	24 (20.3)	0 (0.0)	1 (0.8)	1 (0.8)
**Females (n=108)**	24 (22.2)	1 (0.9)	3 (2.8)	2 (1.9)	1 (0.9)	1 (0.9)
**Total (n=226)**	87 (38.5)	39 (17.3)	27 (11.9)	2 (0.9)	2 (0.9)	2 (0.9)

Overall, 226 students completed the questionnaire with a response rate of 90.4%. The age of the students ranged from 18 to 26 years with median age being 22.0 (Interquartile Range=3). (Table 2)

**Table 2:** Socio demographic profile of the respondents (n= 226).

Categories	n (%)
**Gender**	
Males	118 (52.2)
Females	108 (47.8)
**Year of study**	
Second year	82 (36.3)
Third year	144 (63.7)
**Current place of stay**	
Hostel/Flat	178 (78.8)
Family home	48 (21.2)
**Academic performance**	
Good	61 (27.0)
Average	147 (65.0)
Poor	18 (8.0)

Among the current users of alcohol, 63 (72.4%) were males and 24 (27.6%) were females; current alcohol use being higher in males. Majority of current alcohol users reported using it 1-5 days in the last 30 days, the prevalence being 58 (66.7%), whereas 19 (21.8%) of the current alcohol users were found to be using it 20 or more days in the last 30 days. Although the percentage of females currently using alcohol was lesser than males, the percentage of those taking alcohol 20 or more days in the last 30 days was 33.3% which is higher compared to males, which was 17.5% (Table 3).

**Table 3:** Current use of alcohol, tobacco and cannabis among the respondents.

	Current users	1-5 days in the last 30 days	6-19 days in the last 30 days	20 or more days in the last 30 days
	n	n (%)	n (%)	n (%)
**Alcohol**	87	58 (66.7)	10 (11.5)	19 (21.8)
**Gender**				
Males	63	43 (68.3)	9 (14.3)	11 (17.5)
Females	24	15 (62.5)	1 (4.2)	8 (33.3)
**Year of Study**				
Second year	27	11 (40.7)	4 (14.8)	12 (44.4)
Third year	60	47 (78.3)	6 (10.0)	7 (11.7)
**Start of alcohol use**				
Before joining medical school	49	33 (67.3)	4 (8.2)	12 (24.5)
After joining medical school	38	25 (65.8)	6 (15.8)	7 (18.4)
**Tobacco**	39	17 (43.6)	7 (18.0)	15 (38.4)
**Gender**				
Males	38	17 (44.7)	6 (15.8)	15 (39.5)
Females	1	0 (0.0)	1 (100.0)	0 (0.0)
**Year of Study**				
Second year	14	5 (35.7)	4 (28.6)	5 (35.7)
Third year	25	12 (48.0)	3 (12.0)	10 (40.0)
**Start of tobacco use**				
Before joining medical school	14	5 (35.7)	2 (14.3)	7 (50.0)
After joining medical school	25	12 (48.0)	5 (20.0)	8 (32.0)
**Cannabis**	27	21 (77.8)	3 (11.1)	3 (11.1)
**Gender**				
Males	24	18 (75.0)	3 (12.5)	3 (12.5)
Females	3	3 (100.0)	0 (0.0)	0 (0.0)
**Year of Study**				
Second year	9	7 (77.8)	0 (0.0)	2 (22.2)
Third year	18	14 (77.8)	3 (16.7)	1 (5.6)
**Start of cannabis use**				
Before joining medical school	7	4 (57.1)	1 (14.3)	2 (28.6)
After joining medical school	20	17 (85.0)	2 (10.0)	1 (5.0)

Among the current tobacco users, 38 (97.4%) were males and 15 (39.5%) of those reported using tobacco 20 or more days in the last 30 days. Only one female reported current use of tobacco. Among the current tobacco users, 14 (35.9%) started using it before joining medical school, whereas 25 (64.1%) started using it after joining medical school (Table 3).

Twenty four (88.9%) of current cannabis users were males. Among the males, 18 (75.0%) of them reported consuming cannabis 1-5 days in the last 30 days. Three females reported current use of cannabis, and all of them reported using it 1-5 days in the last 30 days. Among the current cannabis users, 20 (74.1%) started using it after joining medical school (Table 3).

## DISCUSSION

In the present study, the total number of respondents was 226. Forty-two percent of all respondents reported current use of one or more psychoactive substances among which 74.7% were males and 25.3% were females. Prevalence of current use of substance among male respondents was 60.2% and among female respondents was 22.2%. This finding is similar to an earlier study done by Budhathoki et al^7^ among medical students in Nepal which showed that the prevalence of substance use was 49.6%. Another study conducted by Makanjuola et al^8^ done among Nigerian medical students, reported that 40.4% of all respondents were currently using one or more substances; current use among male and female respondents was 44% and 33% respectively. There seems to be a difference in prevalence rate of substance use among males and females, not only in our study but in many similar studies conducted in the past.^2,5,7-8^ This difference could be due to relatively greater social acceptability of substance use among males including other gender norms.^9^

In our study, the most frequently used substance (both lifetime and current use) was alcohol with current use prevalence of 38.5% followed by tobacco (17.3%). Similar findings were reported in studies conducted in Nepal in the past.^2,7^ This could be due to easier accessibility, affordability and minimal legal hindrances involving alcohol and tobacco compared to other substances.^10^ Moreover, alcohol is also used as a part of social and cultural practice among various ethnic groups in Nepal.^11^ Alcohol is extensively served in gatherings and festivals in different ethnic communities, and home brewing of alcohol is also fairly common.^12-13^ This coupled with limited legal regulations regarding the use and sales of alcohol could also be one of the factors that have led to its increased use among medical students in Nepal.^14^

According to the National Institute on Alcohol Abuse and Alcoholism (NIAAA), up to three drinks on a single day and up to seven drinks per week have been defined as low-risk drinking in women. For men, up to four drinks on a single day and up to 14 drinks per week is defined as low-risk drinking.^15^ Fortunately, in our study, a majority of current alcohol users were using it for only 1-5 days in the last 30 days. This although, not very concerning could pose a future risk of increased use of alcohol and therefore shouldn't be dismissed.

Five-point one percent of the global burden of disease has been attributed to the harmful use of alcohol. Furthermore, according to WHO, alcohol is considered to be the major risk factor for premature death and disability among people aged 15 to 49.^16^ In our study, 21.8% of current alcohol users were using it more than 20 days in the last 30 days which could be indicative of harmful drinking and is therefore concerning.

The current use of alcohol was found to be higher in males than females. Besides psychosocial factors, the gender difference in alcohol metabolism could also be a reason for this. Alcohol metabolism in males is more efficient than in females because males have highly active forms of Alcohol Dehydrogenase (ADH) in the stomach and liver.^17^ Females also tend to have lower body mass and a higher proportion of fat to muscle. Thus, females have a higher concentration of alcohol in their blood than adult men with the same amount of alcohol intake.^18^ Even with significantly lower alcohol exposure, females can have alcohol-related physical illnesses and can display more severe cognitive and motor impairment.^19^ In our study, there was a higher percentage of females consuming alcohol 20 or more days in the last 30 days compared to males, which is a significant finding.

Among the total current cannabis users, our study showed that the majority (74.1%) started using it after joining medical school. A similar finding was noted in research done by Budhathoki et al^7^ which showed that all of the cannabis users started using it after joining medical school.

Although the frequency of substance use has been clearly presented, the exact quantification of the amount of substance used couldn't be assessed, which remains a limitation of our study. This study was also conducted on a single institution on a limited number of students, so the results cannot be generalized, and since this study is cross-sectional, a causal relationship could not be established. Therefore, we recommend longitudinal study to be carried out with a larger group of students involving multiple institutions.

## CONCLUSIONS

Almost half of the students in our study were currently using one or more substances, with male predominance. Continued use can have implications on their academic performance and health. Therefore, strategies must be adopted to limit the use of substance, some of which include raising awareness regarding the ill effects of substance use beginning from the first year, imposing and strengthening rules against the use and sale of easily available substances like tobacco and alcohol around college and hospital premises, encouraging engagement in extra-curricular activities like sports, which can serve as a break from the daily stress of student life and also setting up screening facilities within medical colleges.

## References

[1] World Health Organization (WHO) (2021). Drugs (psychoactive) [Internet].

[2] Khanal P, Ghimire RH, Gautam B, Dhungana SK, Parajuli P, Jaiswal AK (2010). Substance use among medical students in Kathmandu valley. J Nepal Med Assoc.

[3] World Health Organization (WHO) (2004). Neuroscience of Psychoactive Substance Use and Dependence. Addiction.

[4] Rai D, Gaete J, Girotra S, Pal HR, Araya R (2008). Substance use among medical students: Time to reignite the debate?. Natl Med J India.

[5] Arora A, Kannan S, Gowri S, Choudhary S, Sudarasanan S, Khosla PP (2016). Substance abuse amongst the medical graduate students in a developing country. Indian J Med Res.

[6] Smart RG, Hughes PH, Johnson LD, Anumonye A, Khant U, Medina Mora ME (1980). A methodology for student drug-use surveys. World Heal Organ Offset Publ.

[7] Budhathoki N, Shrestha MK, Acharya N, Manandhar A (2010). Substance use among third year medical students of Nepal. J Nepal Health Res Counc.

[8] Makanjuola AB, Daramola TO, Obembe AO (2007). Psychoactive substance use among medical students in a Nigerian university. World Psychiatry.

[9] Tsvetkova LA, Antonova NA (2013). The prevalence of drug use among university students in St. Petersburg, Russia. Psychol Russ State Art.

[10] Jaiswal HS, Jain SL, Jaiswal SS (2017). Patterns of Substance Use in First Year and Final year Medical Students: A Cross-sectional Study. Int J Recent Surg Med Sci.

[11] Thapa N, Aryal KK, Puri R, Shrestha S, Shrestha S, Thapa P (2016). Alcohol consumption practices among married women of reproductive age in Nepal: A population based household survey. PLoS One.

[12] Adhikari TB, Rijal A, Kallestrup P, Neupane D (2019). Alcohol consumption pattern in western Nepal: Findings from the COBIN baseline survey. BMC Psychiatry.

[13] Thapa N, Aryal KK, Paudel M, Puri R, Thapa P, Shrestha S (2015). Nepalese Homebrewed Alcoholic Beverages: Types, Ingredients, and Ethanol Concentration from a Nation Wide Survey. J Nepal Health Res Counc.

[14] Dhimal M, Bista B, Bhattarai S, Dixit LP, Hyder MKA, Agrawal N (2020). Report of Non Communicable Disease Risk Factors STEPS Survey Nepal 2019.

[15] Alcohol Research: Current Reviews Editorial Staff. (2018). Drinking Patterns and Their Definitions. Alcohol Res.

[16] World Health Organization. (2018). Global status report on alcohol and health 2018: executive summary.

[17] Edenberg HJ (2007). The genetics of alcohol metabolism: Role of alcohol dehydrogenase and aldehyde dehydrogenase variants. Alcohol Research and Health.

[18] WHO Department of Gender, women and Health. (2005). Gender, health and alcohol use [Internet].

[19] Ceylan-Isik AF, McBride SM, Ren J (2010). Sex difference in alcoholism: Who is at a greater risk for development of alcoholic complication?. Life Sciences.

